# Tunable Superconducting Cavity using Superconducting Quantum Interference Device Metamaterials

**DOI:** 10.1038/s41598-019-40891-1

**Published:** 2019-03-15

**Authors:** Samuel Kim, David Shrekenhamer, Kyle McElroy, Andrew Strikwerda, Jacob Alldredge

**Affiliations:** 1 Research and Exploratory Development Department, Johns Hopkins University Applied Physics Laboratory, Laurel, MD 20723 USA; 2 Present Address: Electrical Engineering and Computer Science Department, Massachusetts Institute of Technology, Cambridge, MA 02139 USA

## Abstract

Here we consider a tunable superconducting cavity that can be used either as a tunable coupler to a qubit inside the cavity or as a tunable low noise, low temperature, RF filter. Our design consists of an array of radio-frequency superconducting quantum interference devices (rf SQUIDs) inside a superconducting cavity. This forms a tunable metamaterial structure which couples to the cavity through its magnetic plasma frequency. By tuning the resonant frequency of the metamaterial through an applied magnetic flux, one can tune the cavity mode profile. This allows us to detune the cavity initially centered at 5.593 GHz by over 200 MHz. The maximum quality factor approaches that of the empty cavity, which is 4.5 × 10^6^. The metamaterial electromagnetic response is controlled via a low-frequency or dc magnetic flux bias, and we present a control line architecture that is capable of applying sufficient magnetic flux bias with minimal parasitic coupling. Together this design allows for an *in-situ* tunable cavity which enables low-temperature quantum control applications.

## Introduction

Waveguide cavities provide a simple resonant system with their fundamental mode set by the speed of propagation in the cavity and the size of the cavity itself. Tuning cavities is traditionally accomplished by physically changing the size of the cavity, which although effective, results in slow and bulky devices. Other methods of tuning include electronic tuning through varactor diodes^1^ or microelectromechanical systems (MEMS)^2^, and magnetic tuning through ferromagnetic or ferrimagnetic resonators such as yttrium iron garret (YIG)^3^. These all have various tradeoffs in terms of switching speed, power handling, tuning range, susceptibility to vibration/noise, and other factors^4^. However, none of these systems are capable of operating at low temperatures. In order to overcome these limitations and create a device useful for cavity control in cryogenic systems, we have designed an electronically tuned cavity that can be rapidly switched between states and is compatible with cryogenic temperatures. This will enable novel cryogenic tunable filters as well as unique cavities that enable coupling control to and between superconducting qubits.

In order to accomplish this tunability in a manner that can operate below 4 K, we use metamaterial rf SQUID arrays inside the cavity to perturb the cavity modes. Metamaterials are artificially designed materials that consist of sub-wavelength unit cells, or meta-atoms, and achieve effective electromagnetic properties that are not available in naturally-occurring materials. We use superconducting metamaterials in order to take advantage of their low losses, high nonlinearity, and ability to probe quantum phenomena. There are several reviews of their use as a low temperature metamaterial available^5,6^. In particular, people have used the nonlinearity of superconductors and Josephson Junctions (JJs) to design tunable superconducting metamaterials. The tuning is provided by either temperature or dc magnetic field and is capable of tuning the resonant frequency or of controlling the electromagnetic transparency^7–15^. Related to these superconducting metamaterials are SQUID metamaterials^7–9^, which rely not on the properties of just superconductors or JJs, but on superconducting SQUIDs made from JJs. Due to the SQUID’s interaction with a magnetic field, they possess an effective uniaxial magnetic permeability that can be dynamically controlled via an applied magnetic flux. In this paper, we use a SQUID metamaterial to design and computationally model a tunable superconducting cavity that demonstrates the high level of control and the compatibility inherent within our proposed design.

Using these SQUID metamaterials inside a cavity, we find that the coupling between the cavity and the metamaterial occurs at the metamaterial’s magnetic plasma frequency, where the effective magnetic permeability *μ*_*r*_ = 0. While there have been recent studies on the behavior of epsilon-near-zero (ENZ) materials in quantum physics, there are relatively few studies of the magnetic equivalent^16,17^. Our work represents a new and novel system for probing the properties of mu-near-zero materials.

Our tunable cavity could also have applications for quantum computing architectures. JJs^13^ and SQUIDs^18,19^ have previously been used to build planar tunable superconducting cavities. Additionally, there has been significant progress with regards to controlling planar qubits by using either a tunable coupler^20^ or cavity^21,22^ to mediate coupling with a qubit. Instead of using a physical tunable cavity or coupler, our device provides a new method of controlling this cavity-qubit coupling by tuning the cavity mode itself, allowing for the coupling and isolation of the qubit. This would allow the qubit to be addressed when the resonance is coupled to the qubit and isolated when it is not, allowing processing to occur during the isolation, where the qubit is insulated from outside noise improving its lifetime.

## Results

### SQUID metamaterial structure and theory

A SQUID metamaterial is composed of rf SQUIDs arranged in a periodic 2D array, each of which function as a meta-atom. These rf SQUIDs are composed of a single JJ in a superconducting loop, and can be effectively modeled as an RLC circuit in series with a variable inductance. We briefly review the rf SQUID model here, but additional details can be found in the literature^8,9^.

The Josephson effect produces a current across the JJ, $I={I}_{c}\,\sin \,\delta (t)$, where *I*_*c*_ is the critical current of the junction, and $\delta$ is the Josephson phase around the superconducting loop. Here the junction has an effective inductance ${L}_{JJ}=\frac{{{\rm{\Phi }}}_{0}}{2\pi {I}_{c}\,\cos \,\delta }$, where ${{\rm{\Phi }}}_{0}=\frac{h}{2e}$ is the flux quantum, *h* is Planck’s constant, and *e* is the elementary charge. The pads of the junction give it a capacitance *C*, any loss in the circuit can be taken into account by a resistance *R*, and the overall geometry of the circuit adds an additional inductance *L*. Hence, the resonant frequency of the SQUID is given by

1 $${\omega }_{0}=\frac{1}{2\pi \sqrt{{(\frac{1}{L}+\frac{1}{{L}_{JJ}})}^{-1}C}}$$

Since the behavior of the JJ is dependent on $\delta$, the total magnetic flux ${\rm{\Phi }}$ through the SQUID is given by ${{\rm{\Phi }}}_{ext}={\rm{\Phi }}+LI$ where ${{\rm{\Phi }}}_{ext}$ is the externally applied magnetic flux. This relation can be written as the differential equation

2 $${{\rm{\Phi }}}_{dc}+{{\rm{\Phi }}}_{rf}\,\sin \,\omega t=\frac{{{\rm{\Phi }}}_{0}\delta }{2\pi }+L({I}_{c}\,\sin \,\delta +\frac{1}{R}\frac{{{\rm{\Phi }}}_{0}}{2\pi }\frac{d\delta }{dt}+C\frac{{{\rm{\Phi }}}_{0}}{2\pi }\frac{{d}^{2}\delta }{d{t}^{2}})$$

where ${{\rm{\Phi }}}_{dc}$ is the dc flux bias of the applied magnetic field, and ${{\rm{\Phi }}}_{rf}$ and $\omega$ are the amplitude and angular frequency of the applied rf field. This system is analogous to the driven and damped oscillator with nonlinear components. In addition, ${L}_{JJ}$ and ${\omega }_{0}$ can be tuned by an applied magnetic flux. For convenience, we define a normalized dc flux bias ${f}_{dc}={{\rm{\Phi }}}_{dc}/{{\rm{\Phi }}}_{0}$.

In the frequency regime where the size of the meta-atom SQUIDs is sufficiently sub-wavelength, the array of SQUIDs acts like a metamaterial with effective relative permeability

3 $${\mu }_{r}=1+F(\frac{{{\rm{\Phi }}}_{ac}}{{{\rm{\Phi }}}_{rf}\,\sin \,\omega t}-1)$$

where *F* is the 2D filling fraction of the meta-atom SQUIDs in the medium and ${{\rm{\Phi }}}_{ac}$ is the ac flux response of the SQUID^8^. It is important to note that this model does not take into account the coupling between SQUIDs, which may introduce higher-order effects on the behavior of the metamaterial^23,24^.

### Cavity Resonance Tuning

**Figure 1:**
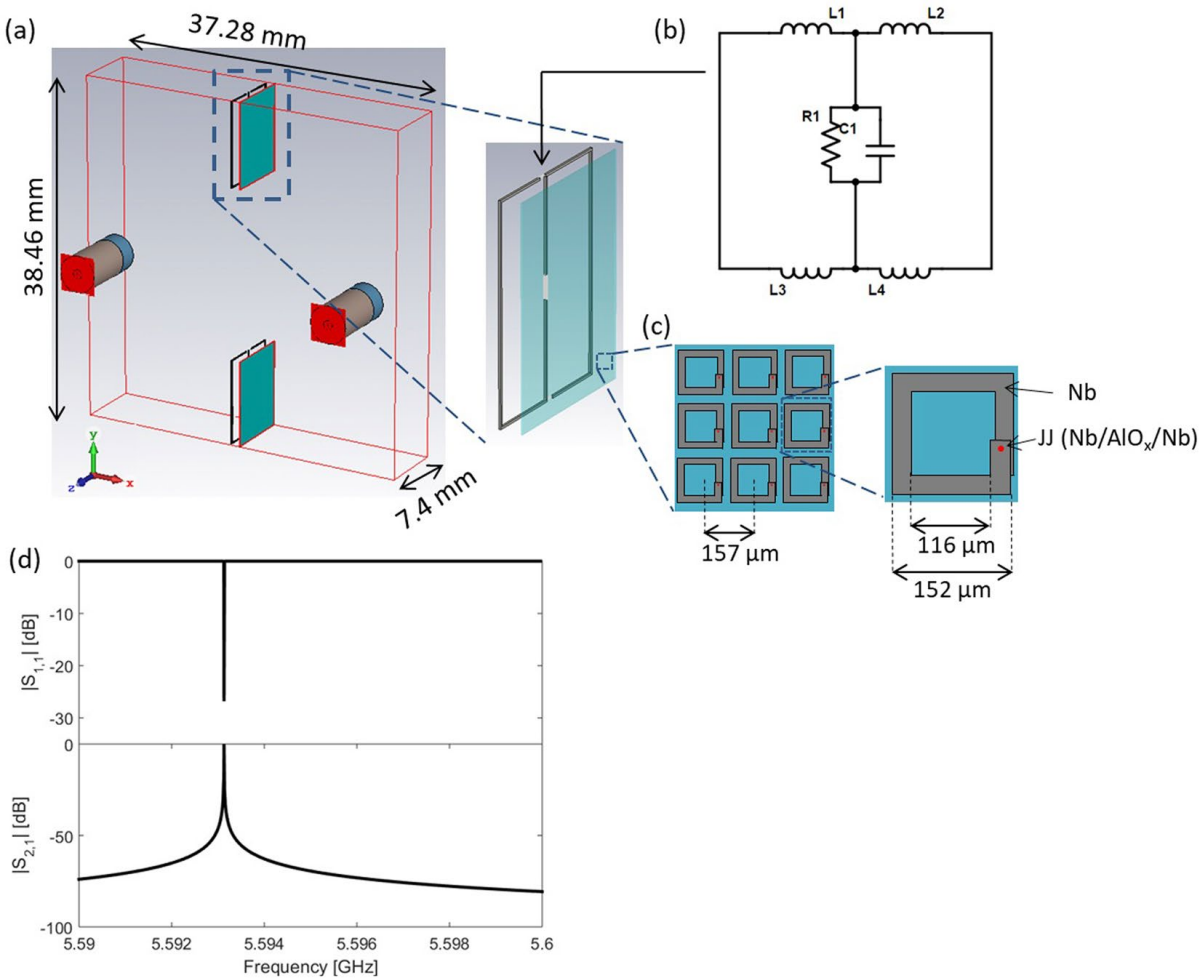
(**a**) CST model of the cavity (outlined in red), the metamaterial (teal), control line (black), SMA pin connectors (tan), and waveguide ports (red squares). The control line is offset from the metamaterial in x, and is designed to tune the metamaterial through an applied magnetic flux. (**b**) A circuit diagram representation of the control line. *R1* is the 50 Ω port, which represents the external power source to drive current in the control line. *L1-4* and *C1* are inductors and a capacitor with values *L* = 10 nH and *C* = 30 pF, respectively, to behave as filters. (**c**) rf-SQUID metamaterial. Geometric parameters have been adjusted to reach the effective RLC circuit parameters described in the Methods section. The rf-SQUID has an overlap of 8700 μm^2^. (**d**) S-parameters for the empty cavity. The resonance is at 5.593 GHz.

The empty cavity we chose for our simulation has a fundamental resonance at 5.593 GHz (see Fig. 1(b)) and a Q of 4.5 × 10^6^. The upper limit on the Q is the result of the impedance of the waveguide ports in and out of the cavity. We place the metamaterial towards the edges of the cavity and extend it halfway towards the center of the cavity, as shown in Fig. 1(a). This positioning maximizes coupling with the magnetic fields of the fundamental mode and minimizes coupling with any potential qubit that may sit at the center of the cavity. While Fig. 1(a) shows the control lines to control the metamaterial, the following results are calculated without the control lines. The control lines will be discussed later on.

**Figure 2:**
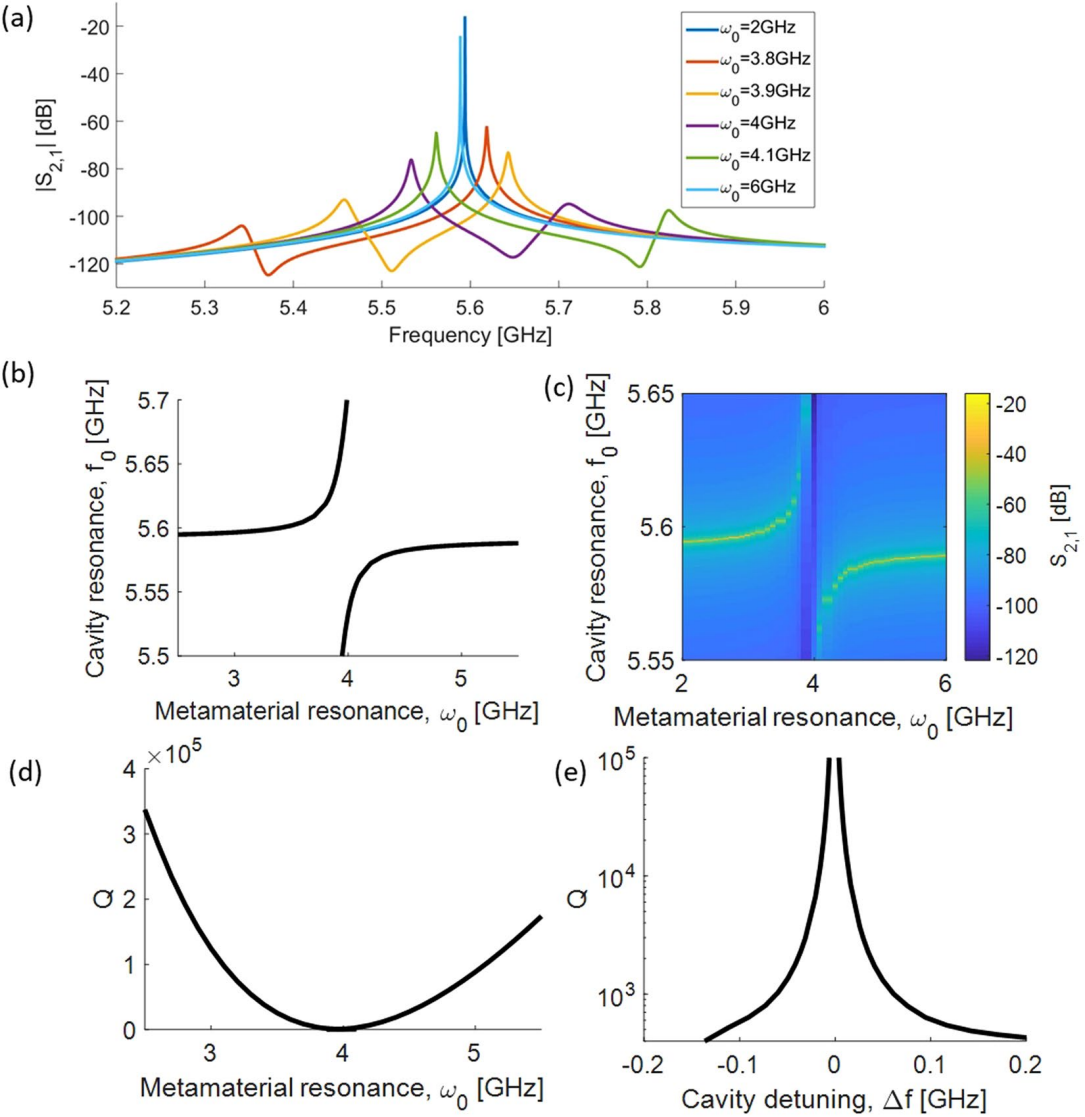
(**a**) *S*2,1 parameter of the cavity for various tunings of the metamaterial. Magnetic permeability damping is ${\rm{\Gamma }}=100\,{\rm{M}}{\rm{H}}{\rm{z}}$. (**b**) Cavity mode as a function of the metamaterial resonance. (**c**) *S*_2,1_ parameter of the cavity as a function of metamaterial tuning and input frequency for a single metamaterial layer. (**d**) Q of the cavity as a function of the metamaterial resonance, for different number of metamaterial layers. (**e**) Q as a function of cavity frequency shift.

Figure 2(a) shows the *S*_2,1_ parameters for various tunings of the metamaterial, ${{\rm{\omega }}}_{0}$. We see peaks of high transmission ranging from 5.3 to 5.9 GHz depending on ${{\rm{\omega }}}_{0}$. These peaks represent the cavity eigenmode, which are plotted in Fig. 2(b). The same data is also shown as an intensity plot in Fig. 2(c).

Looking at the eigenmode in Fig. 2(b), the overall response of the system is similar to that of two coupled oscillators with an avoided crossing. Far from resonance the cavity mode remains unperturbed as the metamaterial is decoupled from the cavity. As they approach resonance, the cavity mode shifts following the equation ${f}_{0}=\frac{{\omega }_{c}+{\omega }_{0}\sqrt{{\mu }_{s}}}{2}\pm \sqrt{{(\frac{{\omega }_{c}-{\omega }_{0}\sqrt{{\mu }_{s}}}{2})}^{2}+{{\rm{\Delta }}}^{2}}$, where ${\omega }_{c}$ is the empty cavity frequency, ${\rm{\Delta }}$ is an effective coupling between the cavity and metamaterial modes, and *μ*_*s*_ is the static magnetic permeability of a Lorentz model fitted to the metamaterial *μ*_*r*_ (details are outlined in the Methods section). Figure 2 shows that we can achieve cavity detuning on the order of 200 MHz close to ${\omega }_{0}=3.95\,{\rm{GHz}}$. In this tuning range, the metamaterial is the most strongly coupled with the cavity and we can observe mode splitting. The behavior of our system fits very well to the coupled oscillator model with a coupling constant of 83 MHz.

Although the cavity resonance is at 5.593 GHz, the metamaterial most strongly couples to the cavity when the metamaterial resonance is tuned to 3.95 GHz, so the coupling does not occur at the metamaterial’s resonance. Instead, we observe maximum coupling and modulation in transmission corresponding to the zero crossing of the metamaterial’s frequency-dependent permeability, which happens when ${\omega }_{c}={\omega }_{0}\sqrt{{\mu }_{s}}$. This is the magnetic plasma frequency at which *μ*_*r*_ = 0 and magneto-inductive waves can propagate^25,26^. Magneto-inductive wave devices have been investigated in waveguide structures^27,28^. However, the nature of this interaction has not been previously observed in any cavity system and although not a focus of this study, is an area of interest for further exploration.

Figure 2(d,e) show the quality factor, Q, of the cavity as a function of the metamaterial tuning. For a single metamaterial layer, Q drops from 10^5^ down to well below 10^3^ as the coupling between the metamaterial and the cavity increases due to the losses in the SQUID. As we further decouple the metamaterial from the cavity, Q approaches that of the empty cavity.

The tuning of the cavity mode provides a mechanism by which one could potentially isolate a qubit or couple to other mode frequencies by modulating the cavity mode. The losses are not necessarily detrimental to such a qubit control scheme if the qubit is tuned to the cavity’s natural resonance with a high Q (>10^5^), and the metamaterial couples to the cavity only for specific, short operations such as fast qubit reset. Additionally, the ability to modulate Q by such a large factor can be used to tune the ring-up or ring-down time of individual qubits. Thus, this system can act as an isolation switch for a qubit allowing many qubits to be addressed in the same frequency by providing rapid on-off addressing.

### Effect of damping parameter

While several of the SQUID parameters are controlled by its geometry and fabrication (*L*, *L*_JJ_), Trepanier *et al*. determine other SQUID parameters (*R*, *C*, *I*_*c*_) are uncertain enough to require fitting in order to determine their value. This fitting is accomplished by measuring *S*_2,1_ and fitting it to the SQUID models^8^. In particular, the effective resistance *R* can only be precisely determined by fitting the width and depth of *S*_2,1_. This fit is subject to the largest uncertainty since it is influenced by the fabrication and measurement quality. Measuring a low Q from the *S*_2,1_ parameter may predict too small of a value for *R*, which in turn would lead to predicting a high value for damping factor ${\rm{\Gamma }}$ in our fitted Lorentz model.

**Figure 3:**
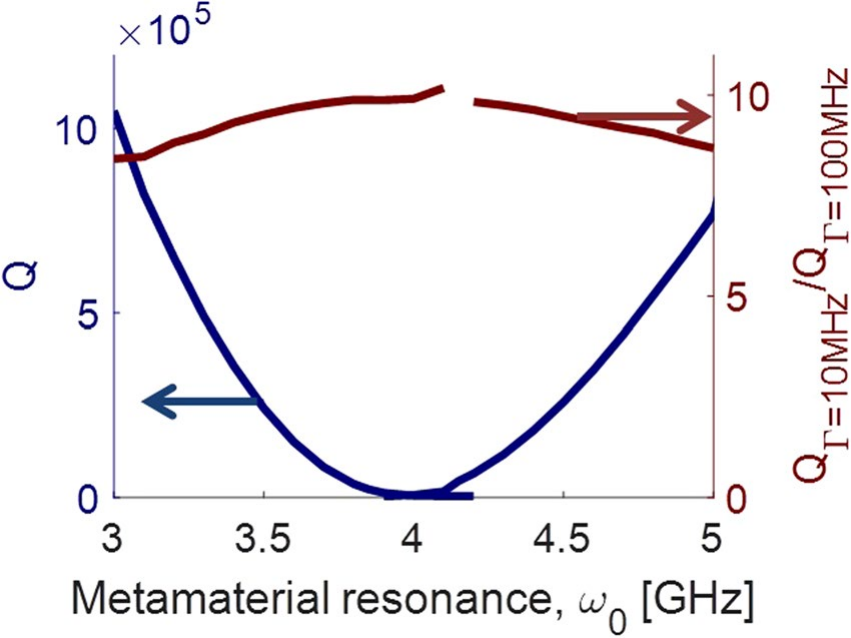
Cavity response as a function of the metamaterial tuning for two different magnetic permeability damping parameters. (left axis) Q of the cavity for ${\rm{\Gamma }}=10\,{\rm{MHz}}$. Q scales approximately inversely with the damping parameter. (right axis) Ratio of Q for ${\rm{\Gamma }}=10\,{\rm{MHz}}$ to Q for ${\rm{\Gamma }}=100\,{\rm{MHz}}$.

Figure 3 shows the quality factor as a function of the metamaterial tuning for a reduced damping factor ${\rm{\Gamma }}=10\,\mathrm{MHz}$. We observe that as ${\rm{\Gamma }}$ is reduced by a factor of 10, then Q increases by a factor of up to 10 at the point of maximum cavity-metamaterial coupling, while cavity detuning remains the same across the range of metamaterial tuning. This can be understood by viewing this as two coupled modes and shows that the mechanism for loss in the cavity mode, when coupled to the metamaterial, is primarily due to losses within the metamaterial. This provides an avenue of improvement for this architecture. If we can reduce the losses in the SQUIDs and substrate, then we can improve the Q and tunable range of the system.

### Control loop for active control of the metamaterial

An important component of our tunable cavity system, is the control mechanism for the SQUID metamaterial. Here, we control the SQUIDs through a tunable applied magnetic flux, which should ideally be uniform across the metamaterial in order to bias the individual SQUIDs equally. Ideally, the control mechanism should generate a significant level of magnetic flux while needing to draw minimum current, thus allowing strong coupling to the metamaterial with minimal heat generated. However, the control also needs to minimize coupling to the cavity and minimize stray magnetic fields in order to reduce direct coupling to a qubit inside the cavity. If this is not the case then the control line could perturb the cavity mode, or the cavity mode might induce undesired currents along the control line that may lead to greater system losses. In addition, the control lines need to be designed around a reasonable control bandwidth in order to allow us to determine what a realistic effective bandwidth of the system could be.

**Figure 4:**
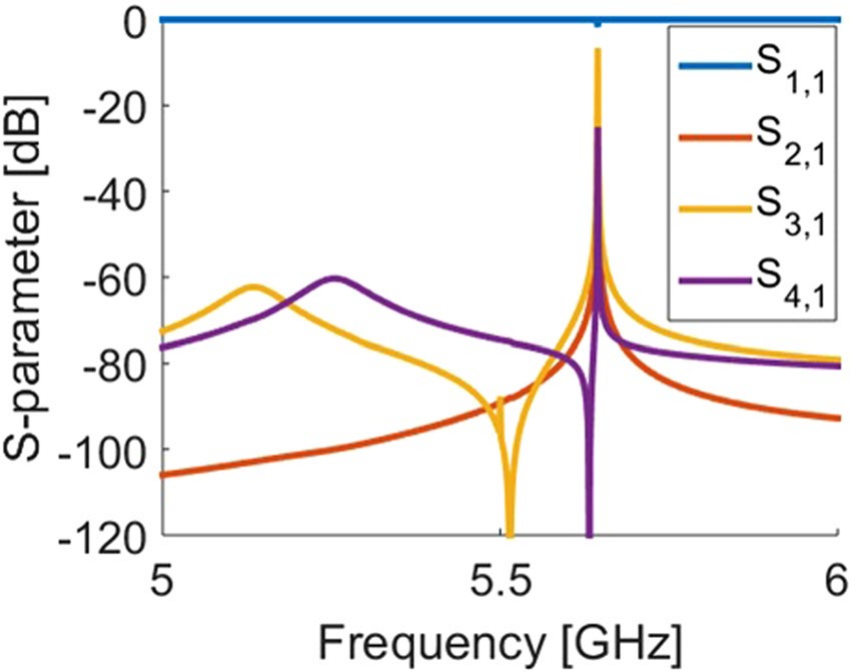
S-parameters of the cavity with the control line, and without the metamaterial. Ports 1 and 2 are the waveguide ports into the cavity, and ports 3 and 4 are the 50 Ω power source for the control lines.

We determined that an appropriate control mechanism design for the system is a superconducting wire in a figure-eight pattern as shown in Fig. 1(a). The superconducting wire is offset from the plane of the metamaterial by a center-to-center distance of 1.05 mm, and it is driven by a 50 Ω port in our CST simulations, which represents the external power source. The magnetic field from the first mode of the cavity induces currents in the loop in opposite directions, cancelling out the driving currents and decoupling the control loops from the cavity. Inductors and capacitors are also introduced into the control line to further minimize coupling with the cavity, and thus, minimize loss through the 50 Ω port. Inductors are placed in series with the port as low-pass filters to block out the high-frequency signals from the cavity. A capacitor is placed in parallel with the port as a high-pass filter. The S-parameters for the cavity with the control line and without the metamaterial is shown in Fig. 4. While there are extra modes introduced, the cavity mode is perturbed only slightly, and maintains a Q of 10^5^. More sophisticated filters could improve this Q by further decreasing cavity-metamaterial coupling.

A caveat of this design is that the two halves of the figure-eight pattern produce magnetic fields in opposite directions. To get around this, note that the SQUID response is approximately symmetric in the applied magnetic flux around *f*_*dc*_ = 0, so the applied magnetic flux will bias the two halves of the metamaterial with equal, but opposite magnitudes. In the small-signal limit where the cavity mode field is much smaller than the applied magnetic flux, the SQUIDs on either half should behave identically, so the effective permeability of the material will be the same.

**Figure 5:**
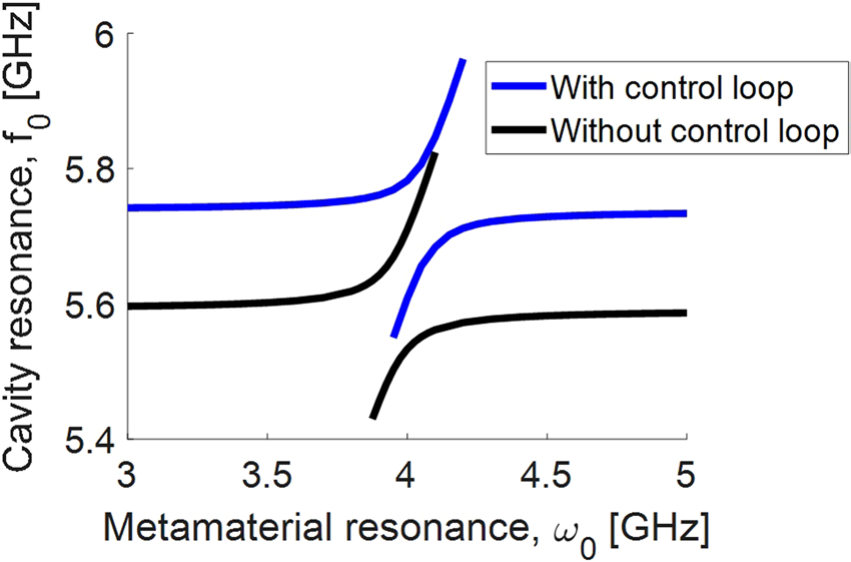
Cavity mode as a function of the metamaterial tuning, where the cavity also contains the control line.

Figure 5 shows the cavity mode frequency as a function of the metamaterial tuning when the figure-eight control loop is inserted into the cavity. We can see that the cavity mode pattern is similar to the results without the control loop, so we still achieve cavity tuning, although the fundamental frequency of the cavity is shifted slightly. By treating the control loop as an RLC circuit, we can also calculate its time constant, $\tau =2.98\,{\rm{ns}}$. The current through the control line thus can be switched very rapidly, enabling fast switching of the cavity mode at 340 MHz. This is much faster than the typical lifetime of a superconducting qubit, which is on the order of 10 s to 100 s of μs^29^.

Future designs will involve placing the control lines in-plane with the SQUIDs, which will reduce fabrication complexity and allow for more complex control line patterns since the control lines do not need to be aligned in a separate fabrication step with the SQUIDs. For example, instead of a quadrupole figure-eight pattern, higher multipole fields can achieve a greater magnetic uniformity across the metamaterial. Another example is to interweave the control lines with the SQUID array in a coplanar geometry, which has the added advantage of uniformly biasing the SQUIDs, providing greater magnetic flux for a given current in the control line, and reducing stray magnetic fields, which would adversely affect a qubit at the center of the cavity.

### Qubit Isolation

This device could be used to control the coupling of a qubit inside the cavity without sacrificing the lifetime of the qubit. To show this, we model a dipole antenna inside the cavity to represent a transmon qubit. While the antenna clearly does not model any of the quantum properties of the qubit, it still models the coupling of the qubit to the cavity.

The dipole antenna contains a 50 Ω port, which is denoted as port 3 (where the waveguide ports are ports 1 and 2). Inductors are placed in series with the port, and physical space between the two arms of the antenna gives rise to capacitance. Thus, we can model the antenna as an RLC circuit whose resonance can be tuned by tuning the inductance. The antenna is placed in the middle of the cavity with the arms aligned along the short dimension of the cavity such that it couples most effectively to the electric field of the cavity’s fundamental mode.

**Figure 6:**
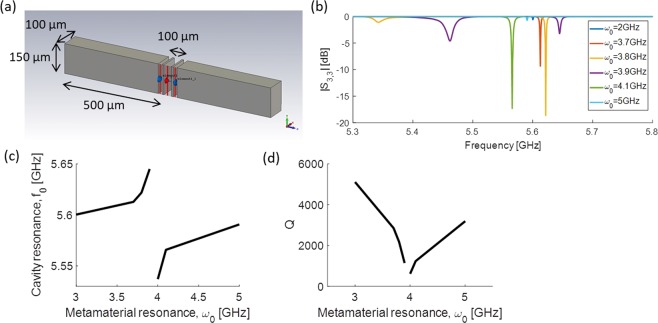
(**a**) Diagram of the dipole antenna that represents a qubit inside the cavity. (**b**) *S*_3,3_ parameter for various tunings of the metamaterial. (**c**) *S*_3,3_ resonance frequency of the qubit as a function of metamaterial tuning. (**d**) Effective Q of the qubit.

We sweep the metamaterial resonance and measure the *S*_3,3_ parameter to see how the qubit couples to the cavity as a function of the cavity mode. The results are shown in Fig. 6. As expected, the qubit exhibits a sharp resonance peak that tracks the resonance of the cavity.

We calculate the effective Q of the qubit for a particular tuning of the metamaterial by $Q={f}_{q}/{{\rm{BW}}}_{q}$ where *f*_*q*_ is the resonance frequency from the *S*_3,3_ parameter and ${{\rm{BW}}}_{q}$ is the 3 dB bandwidth of $\sqrt{1-{S}_{3,3}^{2}}$. The Q is plotted as a function of metamaterial tuning in Fig. 6(d).

In the regime where the metamaterial is most closely coupled with the cavity, the qubit resonance is detuned by 10 s to 100 s of MHz, and the effective Q of the qubit drops allowing fast coupling in and out of the qubit state. However, in this regime, the *S*_3,3_ parameter approaches 1 and the qubit does not couple effectively into the cavity. Away from this regime, the metamaterial is decoupled from the cavity and the qubit effective Q increases to >5,000. The qubit-cavity system is isolated so that they do not see losses through the metamaterial or waveguide ports leaving the qubit isolated for information storage.

## Discussion

In summary, we have designed and computationally modeled a tunable superconducting cavity architecture using a metamaterial consisting of rf SQUIDs. The metamaterial displays an effective magnetic permeability, which is tuned through an applied magnetic flux. By tuning the metamaterial and coupling the metamaterial with the cavity fundamental mode, the cavity mode frequency can be shifted by 100 s of MHz. While the Q of the cavity drops down to 10^3^ for a detuning of 50 MHz, the Q of the detuned cavity can be improved by reducing losses in the SQUIDs.

While temperature dependence of material properties is critical in low-temperature physics, we do not expect temperature dependence of SQUID parameters to be relevant to applications of our cavities to quantum information applications. When the temperature of the system is a significant fraction of the critical temperature of the SQUID materials, such as in Trepanier *et al*.^8^, then the effects are quite large. But for quantum information applications typical temperatures are much lower, 25–50 mK instead of 6.5 K, where SQUID behavior is no longer strongly temperature dependent. For other applications at higher temperatures the tuning range would be lowered and as seen in other experiments..

Further study into the effects of inhomogeneity of the control field is required, as the control mechanism proposed here would not bias the metamaterial uniformly. The dominant effects are expected to be broadening of the metamaterial resonance, which would broaden the coupled cavity mode and decrease the level of detuning. Additional engineering of the control loop can reduce this effect. Additionally, engineering of the SQUIDs to increase the coupling between them or decrease their dc flux sensitivity can further reduce the effect of the field inhomogeneity^23^.

## Methods

In order to design the devices we used finite element method (FEM) simulations of the electromagnetic fields in CST Microwave Studio 2016. Our test chamber is a waveguide cavity resonator with perfect electric conductors (PEC) boundaries that represent the superconducting walls. SMA pin connectors are added to couple the microwave signals in and out of the cavity, and CST waveguide ports are set up at the end of the SMA pin connectors to control input signals and calculate the S-parameters. The dimensions of the cavity are shown in Fig. 1(a).

A metamaterial is embedded in the cavity such that it couples strongly to the magnetic field of the cavity’s fundamental mode. The details of the metamaterial layout and design are described by Trepanier *et al*.^8^. They determined SQUID parameters *L*, *I*_*c*_, and *C* from its geometric design, and $R$ from experimental measurements of *S*_2,1_. We use similar RLC model parameter values, but adjust them to bring the SQUID resonance frequency close to that of the empty cavity. This ensures that we are modeling realistic devices but in the frequency range of interest. The parameters we use are *L* = 0.27 nH, *C* = 2 pF, *R* = 820 Ω, and *I*_*c*_ = 1.2 μA. The thickness of the metamaterial is taken to be the SQUID-to-SQUID separation distance, 157 µm. The geometry of the rf-SQUID shown in Fig. 1 is adjusted to achieve these RLC parameters. Similar to the rf-SQUID of Trepanier *et al*., there are two layers of Nb separated by a 200 nm layer of SiO_2_. The overlap of the two layers gives the capacitance of the rf-SQUID. A Nb/AlO_x_/Nb Josephson Junction connects the two layers.

**Figure 7:**
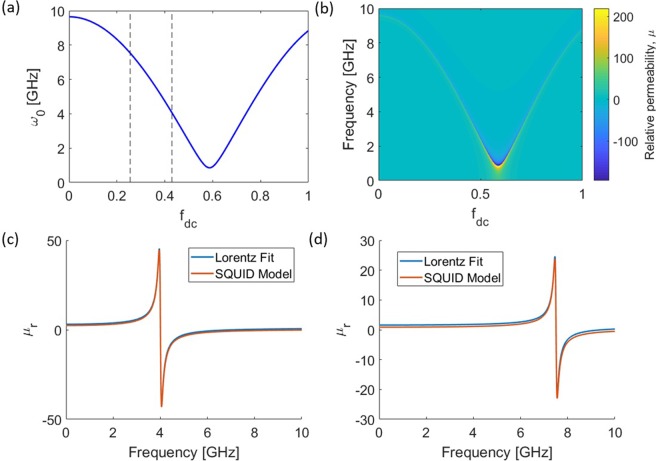
(**a**) SQUID resonance as a function of applied magnetic flux. *L* = 0.27 nH, *C* = 2 pF, *R* = 820 Ω, and *I*_*c*_ = 1.2 μA. (**b**) Effective magnetic permeability, *μ*, as a function of dc bias of the applied magnetic flux *f*_*dc*_ and frequency of the applied magnetic field. We use a filling fraction $F=\frac{{\boldsymbol{\pi }}{40}^{2}}{{83}^{2}}$. (**c**,**d**) Effective magnetic permeability for 2 different values of ${{\rm{f}}}_{{\rm{d}}{\rm{c}}}$ marked in part (**a**,**c**) *μ*_*s*_ = 3.13, ${{\mu }}_{\infty }=1$, ${\omega }_{0}=4.03{\rm{GHz}}$, ${\rm{\Gamma }}=100{\rm{MHz}}$. (**d**) ${{\mu }}_{{\rm{s}}}=1.66$, ${{\mu }}_{\infty }=1$, ${\omega }_{0}=7.06{\rm{GHz}}$, ${\rm{\Gamma }}=91.5{\rm{MHz}}$.

Figure 7 shows plots of ${{\rm{\omega }}}_{0}$ and ${{\rm{\mu }}}_{{\rm{r}}}$ as a function of ${{\rm{f}}}_{{\rm{dc}}}$, which are calculated using Eq. (1–3). Normally we expect the periodicity of ${{\rm{\omega }}}_{0}$ to be *f*_*dc*_ = 1, but this is only the case when the mean ${{\rm{\omega }}}_{0}$ is much greater than the modulation in ${{\rm{\omega }}}_{0}$. Since the mean and modulation of ${{\rm{\omega }}}_{0}$ are comparable in our case, the periodicity is slightly greater than 1.

To simplify the simulation, the metamaterial is replaced in CST by a uniform, dispersive material with a uniaxial magnetic permeability and unity relative permittivity. The unit-cell dimension of the SQUIDs is very sub-wavelength (*λ*/646), so this is a valid approximation in line with effective medium theory. Here we use the Lorentz model for the effective permeability:

4 $${\mu }_{r}(\omega )={\mu }_{\infty }+\frac{({\mu }_{s}-{\mu }_{\infty }){\omega }_{0}^{2}}{{\omega }_{0}^{2}+i\omega {\rm{\Gamma }}-{\omega }^{2}}$$

where ${\mu }_{\infty }$ and *μ*_*s*_ are the infinite and static magnetic permeabilities, respectively, ${\omega }_{0}$ is the aforementioned resonant frequency, and ${\rm{\Gamma }}$ is the damping factor. These parameters are determined by fitting the Lorentz model to the effective relative permeability calculated by Eq. 3. The fits of *μ*_*r*_ to the Lorentz model are shown in Fig. 7(c,d).

As shown in Fig. 1, the metamaterial extends across the depth of the cavity and a quarter ways along the length. Thus, each side of the metamaterial is 9.62 × 7.4 mm^2^, giving our device a total of 20,656 SQUIDs.

The substrate of the SQUID array is not modeled directly, but the losses from the substrate were taken into account through *R* when extracting the effective parameters of the SQUIDs. Additionally, due to the substrate being electrically thin, the permittivity has a negligible effect on the cavity mode and is thus ignored in our model. When modeling the flux tuning of the metamaterial in CST, we assume for simplicity that only ${\omega }_{0}$ is changing while the other parameters remain constant. We can see from fitting *μ*_*r*_ in Fig. 7(c,d) that ${\rm{\Gamma }}$ and *μ*_*s*_ do not change significantly for various values of *f*_*dc*_, so our assumption is reasonable. For all of the simulations, we use magnetic permeability parameters ${\mu }_{\infty }=1$, and *μ*_*s*_ = 2. Unless otherwise stated, ${\rm{\Gamma }}=100{\rm{MHz}}$.

## References

[1] Hunter IC, Rhodes JD (1982). Electronically Tunable Microwave Bandpass Filters. IEEE Trans. Microw. Theory Tech..

[2] Liu X, Katehi LPB, Chappell WJ, Peroulis D (2010). High-$Q$ Tunable Microwave Cavity Resonators and Filters Using SOI-Based RF MEMS Tuners. J. Microelectromechanical Syst..

[3] Carter PS (1961). Magnetically-Tunable Microwave Filters Using Single-Crystal Yttrium-Iron-Garnet Resonators. IEEE Trans. Microw. Theory Tech..

[4] Uher J, Hoefer WJR (1991). Tunable microwave and millimeter-wave band-pass filters. IEEE Trans. Microw. Theory Tech..

[5] Anlage SM (2011). The physics and applications of superconducting metamaterials. J. Opt..

[6] Jung P, Ustinov AV, Anlage SM (2014). Progress in superconducting metamaterials. Supercond. Sci. Technol..

[7] Butz S, Jung P, Filippenko LV, Koshelets VP, Ustinov AV (2013). A one-dimensional tunable magnetic metamaterial. Opt. Express.

[8] Trepanier M, Zhang D, Mukhanov O, Anlage SM (2014). Realization and Modeling of Metamaterials Made of rf Superconducting Quantum-Interference Devices. Phys. Rev. X.

[9] Lazarides, N. & Tsironis, G. P. Rf superconducting quantum interference device metamaterials. *Appl*. *Phys*. *Lett*. **90** (2007).

[10] Jung P, Butz S, Shitov SV, Ustinov AV (2013). Low-loss tunable metamaterials using superconducting circuits with Josephson junctions. Appl. Phys. Lett..

[11] Kurter C, Lan T, Sarytchev L, Anlage SM (2015). Tunable Negative Permeability in a Three-Dimensional Superconducting Metamaterial. Phys. Rev. Appl..

[12] Zhang D, Trepanier M, Mukhanov O, Anlage SM (2015). Tunable Broadband Transparency of Macroscopic Quantum Superconducting Metamaterials. Phys. Rev. X.

[13] Zueco D (2013). From Josephson junction metamaterials to tunable pseudo-cavities. Supercond. Sci. Technol..

[14] Ustinov AV (2014). Experiments With Tunable Superconducting Metamaterials. IEEE Trans. Terahertz Sci. Technol..

[15] Maimistov AI, Gabitov IR (2010). Nonlinear response of a thin metamaterial film containing Josephson junctions. Opt. Commun..

[16] Liberal I, Engheta N (2016). Nonradiating and radiating modes excited by quantum emitters in open epsilon-near-zero cavities. Sci. Adv..

[17] Liberal I, Engheta N (2017). Zero-index structures as an alternative platform for quantum optics. Proc. Natl. Acad. Sci. USA.

[18] Palacios-Laloy A (2008). Tunable Resonators for Quantum Circuits. J. Low Temp. Phys..

[19] Sandberg M (2008). Tuning the field in a microwave resonator faster than the photon lifetime. Appl. Phys. Lett..

[20] Chen Y (2014). Qubit architecture with high coherence and fast tunable coupling. Phys. Rev. Lett..

[21] Blais A, Huang RS, Wallraff A, Girvin SM, Schoelkopf RJ (2004). Cavity quantum electrodynamics for superconducting electrical circuits: An architecture for quantum computation. Phys. Rev. A - At. Mol. Opt. Phys..

[22] Whittaker JD (2014). Tunable-cavity QED with phase qubits. Phys. Rev. B - Condens. Matter Mater. Phys..

[23] Trepanier M (2017). Coherent Oscillations of Driven rf SQUID Metamaterials. Phys. Rev. E.

[24] Tsironis GP, Lazarides N, Margaris I (2014). Wide-band tuneability, nonlinear transmission, and dynamic multistability in SQUID metamaterials. Appl. Phys. A Mater. Sci. Process..

[25] Pendry JB, Holden AJ, Robbins DJ, Stewart WJ (1999). Magnetism from conductors and enhanced nonlinear phenomena. IEEE Trans. Microw. Theory Tech..

[26] Shamonina E, Kalinin VA, Ringhofer KH, Solymar L (2002). Magnetoinductive waves in one, two, three dimensions. J. Appl. Phys..

[27] Freire MJ, Marqués R, Medina F, Laso MAG, Martin F (2004). Planar magnetoinductive wave transducers: Theory and applications. in. Applied Physics Letters.

[28] Syms RRA, Young IR, Solymar L (2006). Low-loss magneto-inductive waveguides. J. Phys. D. Appl. Phys..

[29] Wendin G (2017). Quantum information processing with superconducting circuits: a review. Reports Prog. Phys..

